# An update on the role of *Atopobium vaginae* in bacterial vaginosis: what to consider when choosing a treatment? A mini review

**DOI:** 10.1007/s00404-019-05142-8

**Published:** 2019-04-05

**Authors:** Werner Mendling, Ana Palmeira-de-Oliveira, Stephan Biber, Valdas Prasauskas

**Affiliations:** 1 German Center for Infections in Obstetrics and Gynaecology, Wuppertal, Germany; 2 Labfit-HPRD: Health Products Research and Development, Lda, Covilhã, Portugal; 3 CICS-UBI: Health Sciences Research Centre, University of Beira Interior, Covilhã, Portugal; 4 Scientific and Medical Department, Medinova AG, Zurich, Switzerland

**Keywords:** Bacterial vaginosis, Bacterial biofilm, *Atopobium vaginae*, Metronidazole, Clindamycin, Dequalinium chloride, Microbial resistance

## Abstract

**Introduction:**

*Bacterial vaginosis* (BV) is the most common vaginal disorder in reproductive-age women. The condition is characterised by the replacement of a healthy, lactobacilli-dominated vaginal microbiota by anaerobic and facultative anaerobic bacteria. BV increases the risk of acquisition of STIs and is associated with pregnancy complications. Although the composition of the bacteria in BV varies between individuals, there are some species such as *Gardnerella*, *Atopobium*, *Mycoplasma*, *Snethia*, *Megasphera*, *Dialister*, etc., that are found most frequently.

**Material and Methods:**

Literature research to the importance of *Atopobium vaginae* in BV and treatment options.

**Results:**

* Atopobium (A.) vaginae* is an important component of the complex abnormal vaginal flora in BV; even though *A. vaginae*, like *Gardnerella vaginalis*, has also been detected in the normal flora, it is much more common in BV patients. *A. vaginae* has been shown to play an important role in the pathophysiology of BV and is thought to be at least a partial cause of the known negative sequelae. The presence of *A. vaginae* in the BV-associated biofilms and its resistance to some antimicrobial substances has been described - this seems to have a major impact on treatment outcome.

**Conclusion:**

Current scientific data demonstrate that dequalinium chloride (Fluomycin^®^) is one of the valid therapeutic options for BV treatment, since it displays a broad antimicrobial spectrum against relevant vaginal pathogens, especially against * G. vaginalis* and * A. vaginae*, without having safety concerns.

## Introduction

Bacterial vaginosis (BV) is the most common vaginal disorder in reproductive-age women [1]. The condition is characterised by the replacement of a lactobacilli-dominated vaginal microbiota by anaerobic and facultative anaerobic bacteria. It is still unknown whether the loss of lactobacilli precedes or follows the upheaval of flora in BV [2]. However, it seems to be quite certain that it is a preferential proliferation of the BV-related bacteria, rather than an exogenous acquisition [2].

Only some women with BV have symptoms, displaying a malodorous, watery, grey discharge. Because of the absence of inflammation, BV is not called vaginitis—there is no pain, no itching, no dyspareunia, no redness of the vulva or vagina, and no toxic leucocytes—only a microbial shift to anaerobic pathogens [3]. However, BV increases the risk of acquisition and transmission of STIs [4] and is associated with adverse obstetric and gynaecologic outcomes including miscarriage, premature labour, preterm birth, preterm prelabour rupture of membranes, chorioamnionitis, intrauterine infection, post-caesarean endometritis, upper genital tract infections, and pelvic inflammatory disease [5, 6].

Although the composition of the bacteria in BV varies between individuals, *Gardnerella, Atopobium, Mycoplasma, Prevotella, Bifidobacterium, Megasphaera, Leptotrichia, Sneathia, Dialister, Clostridium,* and Bacterial Vaginosis-Associated Bacterium (BVAB)-1, -2 and -3 species are found most frequently [7, 8]. The association of *G. vaginalis* with BV was originally described by Gardner and Dukes already in 1955 [9]. *G. vaginalis* and *Prevotella spp*. are found in the disturbed vaginal microbiome, but they are also present in lower loads in healthy women [7, 10]. However, the involvement of *A. vaginae* in BV*,* although it rarely occurs in the absence of *G. vaginalis,* has only been established in recent years [11–13]. Increasing evidence on its involvement in BV biofilm formation, as well as on specific resistances of *A. vaginae* against standard antibiotics may explain therapeutic failures and recurrences of BV [14].

Despite the current knowledge on BV many questions regarding treatment remain unanswered—all current therapies have disadvantages and gaps [14]. This mini review describes the current scientific knowledge regarding the relevance of *A. vaginae* in the pathogenesis of BV and what should be considered when choosing a treatment.

## Role of *A. vaginae* in BV

**Fig. 1:**
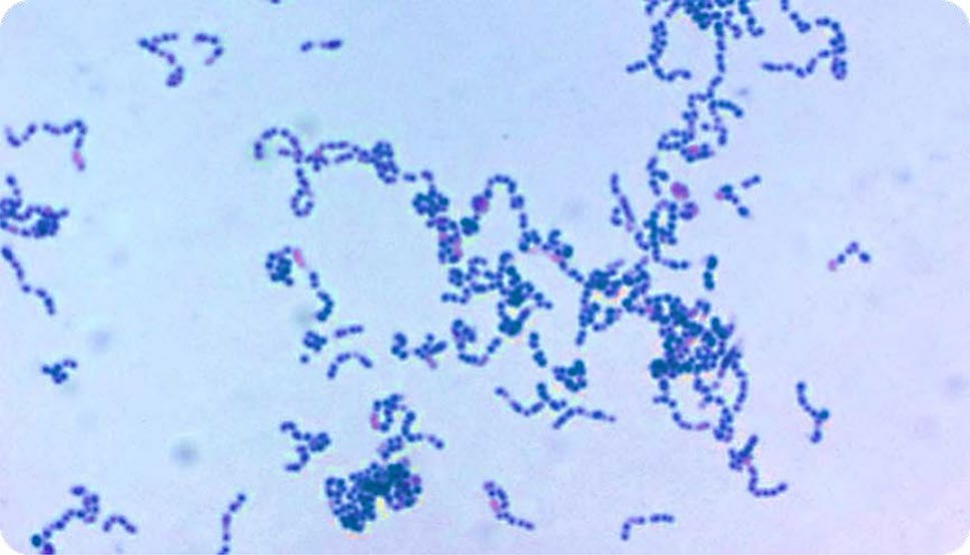
*Atopobium vaginae* culture, Gram stain, magnification 100 × (courtesy of M. Vaneechoutte, Belgium)

*Atopobium vaginae* is a newly discovered bacterium frequently found in women with BV [15]. The name *Atopobium,* meaning “strange living thing” in Greek, was proposed in 1992 [16] to reclassify three bacterial species formerly designated *Lactobacillus minutus, Lactobacillus rimae* and *Streptococcus parvulus.* Genus *Atopobium* belongs to the *Coriobacteriaceae* family and *A. minutum, A. rimae, A. parvulum,* and, later described *A. deltae and A. fossor,* can be distinguished [15]. In 1999 Rodriguez et al. [17] first described *A. vaginae* isolated from the vagina of a healthy women in Sweden. These are Gram-positive, elliptical or rod-shaped cocci, nonmotile and non-spore-forming organisms, and occur alone, in pairs, in clumps or in short chains (Fig. 1). They produce major amounts of lactic acid next to acetic and formic acids and are strictly anaerobic.

Only recently, the association of *A. vaginae* with BV was reported [11, 12] and many subsequent studies have confirmed the bacterium to be an important component of the complex abnormal vaginal flora in BV [11, 12, 18–21]. Even though *A. vaginae*, as *G. vaginalis*, has also been detected in the normal vaginal flora (8% [11] up to 25% [19]), it is found much more commonly in BV patients (50% [12], 55% [11], 83% [18], 96% [19]). Also, the differences between African and Caucasian women, referring the prevalence of *A. vaginae* in the first ones have been described [22]. Loads of *G. vaginalis, A. vaginae,* and other typical BV-pathogens are significantly higher in the BV-positive group than in healthy controls [23]. Additionally, *A. vaginae* has been positively associated with BV typical vaginal discharge, an elevated pH and the presence of clue cells [18, 24]. It was also described that high vaginal loads of *A. vaginae* in combination with *G. vaginalis* are associated with late miscarriage and prematurity [6, 25]. Additionally, it was demonstrated using an in vitro model that *A. vaginae* stimulates an innate immune response from epithelial cells, leading to localised IL-6 and IL-8 and an antimicrobial β-defensin peptide production occurring after triggering the toll-like receptor 2, and this possibly contributes to the pathogenesis of BV [26]. Hence, in BV *G. vaginalis* and *A. vaginae* still belong to ‘the main suspects’, a possible synergism between the two organisms has been considered, and because of this several authors using molecular-based techniques have examined the possibility of combining loads of *A. vaginae* and *G. vaginalis* as a means of diagnosing BV [15, 27, 28].

## Involvement of *A. vaginae* in biofilm formation

Bacteria rarely exist as single-species planktonic forms but thrive in complex polymicrobial adhering communities enveloped by extracellular matrices, so-called biofilms. The bacteria account for less than 10% of biofilm mass, whereas the biofilm matrix usually accounts for more than 90% and provides the best living conditions for the bacteria [29]. Costerton et al. in 1999 [30] have described the association of a bacterial biofilm with various chronic infections. Regardless of the location in the human body, biofilm infections share similar clinical characteristics. They grow slowly, and bacterial communities are rarely fully destroyed by the host-defence mechanisms. Bacteria in biofilm release antigens resulting in an increase in antibody production. However, due to the biofilm structure, the produced antibodies are not capable of killing the biofilm bacteria [30].

The biofilm formation in BV is a virulence mechanism that enhances pathogenicity [31]. The polymicrobial BV-biofilm can be seen with the Gram-stain method in the form of clue cells, which are vaginal epithelial cells covered by layers of adherent Gram-negative and/or -variable cells. Using fluorescence in situ hybridisation (FISH) method, the structure and composition of the biofilm can be studied in more detail [32], especially considering the combined presence of *G. vaginalis* and *A. vaginae*. Hardy et al. [33], similar to that previously described by Swidsinski et al. [31], have demonstrated that adherent *A. vaginae* and *G. vaginalis* were visualised in, respectively, 54% and 82% of samples with bacterial biofilm in BV. It was detected that *G. vaginalis* accounted for 60% or more and *A. vaginae* accounted for 40% or less of the film bacterial composition. It is assumed that *G. vaginalis* acts as an initial coloniser to establish early biofilm structures to which secondary colonisers, such as *A. vaginae* can attach [33, 34]. The fact that *G. vaginalis* is capable of displacing protective lactobacilli on pre-coated vaginal epithelial cells, is probably related to its ability to promote biofilm formation. In contrast, the other anaerobes, including *A. vaginae,* are easily outcompeted by *L. crispatus *[35]. Hardy et al. [33] also demonstrated the important role of *A. vaginae* together with *G. vaginalis* in BV-associated biofilm. Interactions among these species within a biofilm are synergistic: these include co-aggregation, metabolic cooperation and increased resistance to antibiotics or host immune responses and have important clinical implications [36]. The presence of a biofilm—due to increased resistance to treatment—is thought to be one of the possible reasons for the BV recurrence [37, 38].

## Considerations on conventional treatments

Concerning the treatment options, the mainstay of BV treatment in many countries remains either oral or vaginal metronidazole once a day for 5–7 days or vaginal clindamycin as first-line treatments. The efficacy of the treatment with metronidazole is comparable to topical clindamycin [39]. Cure rates, following intravaginal treatment with metronidazole or clindamycin, account for 70–90% at the end of treatment and 1 month after the end of therapy [3, 4, 40]. However, as it was first and best described by Larsson and Forsum in 2005 [39], 3 months after the treatment the recurrence rate can exceed 30% [41].

De Backer et al. [42] demonstrated that susceptibility to metronidazole varied significantly across various *A. vaginae* strains in vitro*.* Some of the investigated clinical isolates of *A. vaginae* were shown to be highly resistant to nitroimidazoles such as metronidazole and secnidazole [11, 43] and it was suggested that this could play a role in treatment failure [11, 42]. Susceptibility testing for metronidazole of additional *A. vaginae* isolates demonstrated that the minimum inhibition concentration (MIC) is variable, ranging from 2 µg/mL (sensitive) to more than 256 µg/mL (resistant) [12]. So far more than half of the tested isolates were resistant.

Beigi and colleagues described a significant increase of clindamycin-resistant anaerobic bacteria after treatment [44]. It is unclear if this is true for *A. vaginae* specifically. Even though these resistance findings seem to be interesting, it is questionable whether it influences clinical efficacy in patients with recurrent BV formerly treated with clindamycin. In vitro data suggest that clindamycin is effective against *A. vaginae* already at low concentrations [45].

## Exploring alternative approaches

**Fig. 2:**
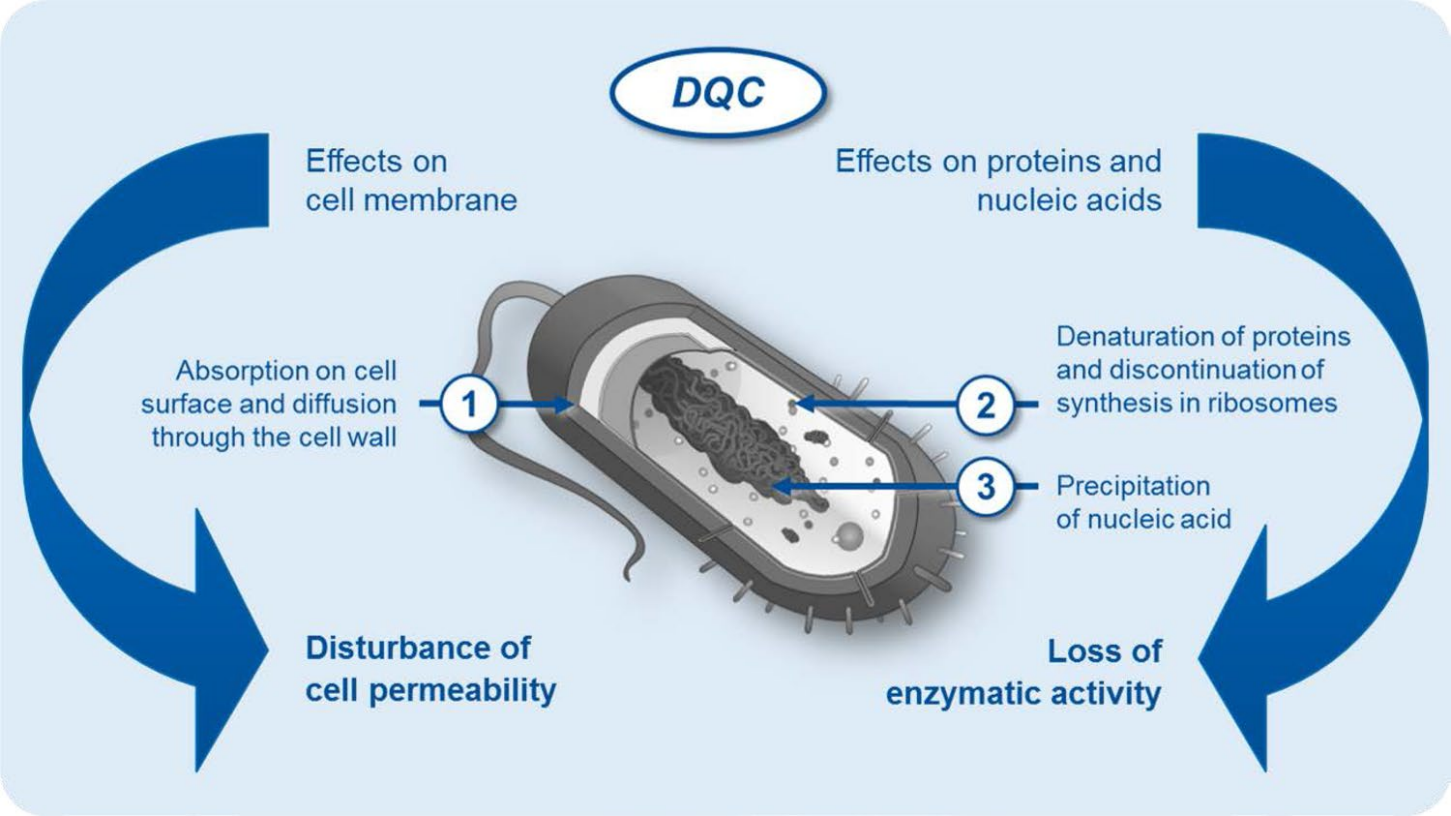
Mode of action of dequalinium chloride [47]. Dequalinium chloride (DQC) acts as a microbicidal against all main vaginal pathogens and due to the multiple modes of action, the development of resistances is unlikely

Alternatives to current antibiotic treatments against BV are increasingly being explored: antiseptics, probiotics, plant-derived compounds, vaginal acidifying and buffering agents, as well as different combination therapies are increasingly used [46–48]. A big interest, due to beneficial characteristics, has been seen in a group of antimicrobial substances belonging to antiseptics, such as dequalinium chloride (DQC) (Fig. 2) [47]. DQC was recently listed in an international guideline as an alternative treatment for BV [49]. Some authors [50] have suggested that DQC, as an antiseptic substance, could be preferable to a repeated course of antibiotics for patients with frequently recurrent BV. The antimicrobial activity of DQC has been investigated and demonstrated over the past decades by several investigators [51–55]. Della Casa et al. [51] has demonstrated the in vitro antimicrobial activity of this substance against different pathogens that are relevant for vaginal infections, including anaerobic bacteria (*G. vaginalis*, etc.), aerobic bacteria (staphylococci, streptococci, etc.), and *Candida* species. Additionally, the non-inferiority of DQC vaginal tablets to clindamycin vaginal cream for the treatment of BV has been demonstrated in a clinical study by Weissenbacher et al. [48]. Based on Amsel’s criteria, the clinical cure rates 4 weeks after the end of treatment with DQC were 79.5% and 77.6% with clindamycin, respectively.

It has been shown that a high concentration of *A. vaginae* before treatment was associated with complete or partial failure of treatment for BV [11, 42]. Additionally, the presence of *A. vaginae* in the BV-associated biofilm has been described [31] and it seems that this knowledge has a major impact on treatment [33].

A recent study by Lopes dos Santos Santiago et al. [45] has investigated the in vitro susceptibility of *A. vaginae* to DQC in comparison to established substances (metronidazole and clindamycin). The MIC (minimal inhibition concentration) and MBC (minimal bactericidal concentration) range of DQC for 28 strains, belonging to 4 species of the genus *Atopobium,* i.e., *A. minutum, A. rimae, A. parvulum,* and *A. vaginae*, have been determined. The MIC and MBC for *Atopobium* spp. to DQC ranged between < 0.0625 and 2 µg/mL with an MIC_90_-value of 2 µg/mL. The MIC_90_-value for *A. vaginae* was demonstrated to be 0.5 µg/mL, i.e., *A. vaginae* was more sensitive than the other species tested. Not only was growth of *A. vaginae* inhibited at the MIC-levels, but the bacterial cells were also killed. The MICs of clindamycin and DQC for *A. vaginae* in this study were similar (sensitive), whereas the MIC of metronidazole was much higher (resistant) [45].

## Biofilm disruption for treatment success

**Fig. 3:**
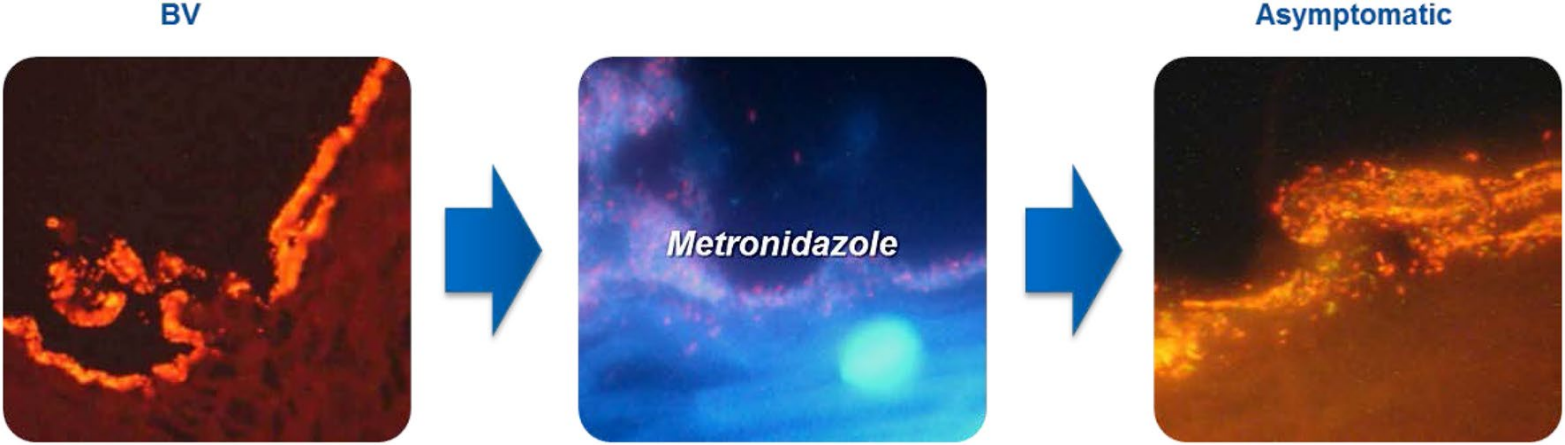
BV-biofilm [31, 38]. A bacterial biofilm, an adhering microbial community enveloped by extracellular matrices, is considered one of the possible reasons for the BV recurrence

Persistence of an adherent bacterial biofilm, containing mostly *G. vaginalis* and *A. vaginae*, can be considered as the major reason for failure of BV treatment (Fig. 3) [31, 38]. Bacteria in biofilms are less susceptible to antibiotics compared to planktonic cells and have a higher tolerance towards antimicrobial treatment [56]. It was found, that although all patients recovered after oral metronidazole treatment, a large reservoir of *A. vaginae* (together with *G. vaginalis*) was persisting as a bacterial biofilm [38]. Additionally, an in vivo study with topical metronidazole gel by Bradshaw et al. [57] found that rates of recurrence of BV were higher when *A. vaginae* was present in addition to *G. vaginalis*. Interestingly, an in vitro study has demonstrated the ability of beneficial *Lactobacillus spp.* to disrupt the biofilm consisting of *A. vaginae* and *G. vaginalis *[58].

In the course of recent in vitro investigations, Gottschick and colleagues have screened various compounds for vaginal Biofilms (consisting of *G. vaginalis*): tThe antibiotics, such as metronidazole and tobramycin were effective in preventing biofilm formation, but had no effect on an established biofilm, while some antiseptic substances led to the disintegration of existing biofilms [59]. Recent not yet published in vitro data are suggesting that DQC could be effective in disrupting the BV-biofilm under experimental conditions [60].

## Conclusions

*A. vaginae* is an important component of the complex abnormal vaginal flora in BV. Resistance of *A. vaginae* to metronidazole, one of the current first-line treatments, as well as the presence of *A. vaginae* in the BV-associated biofilm have been described. Although more clinical data would be needed on this subject, this seems to have a major impact on BV treatment outcomes. Nevertheless, recently available scientific data confirms that DQC (Fluomizin^®^) is one of the valid therapeutic options for BV treatment, as it displays a broad antimicrobial spectrum against relevant vaginal pathogens, especially against *G. vaginalis* and *A. vaginae*, without having safety concerns.
